# Evidence of *in vitro mecB*-mediated β-lactam antibiotic resistance transfer to *Staphylococcus aureus* from *Macrococcus psychrotolerans* sp. nov., a psychrophilic bacterium from food-producing animals and human clinical specimens

**DOI:** 10.1128/aem.01652-24

**Published:** 2025-03-11

**Authors:** Ivana Mašlaňová, Vojtěch Kovařovic, Tibor Botka, Pavel Švec, Ivo Sedláček, Ondrej Šedo, Adéla Finstrlová, Meina Neumann-Schaal, Sarah Kirstein, Sybille Schwendener, Eva Staňková, Kristína Rovňáková, Petr Petráš, Jiří Doškař, Vincent Perreten, Roman Pantůček

**Affiliations:** 1 Department of Experimental Biology, Section of Genetics and Molecular Biology, Faculty of Science, Masaryk University 123906 , Brno, Czech Republic; 2 Institute of Microbiology, Czech Academy of Sciences, BIOCEV, Vestec, Czech Republic; 3 Department of Experimental Biology, Czech Collection of Microorganisms, Faculty of Science, Masaryk University 123906 , Brno, Czech Republic; 4 Central European Institute of Technology, Masaryk University 622272 , Brno, Czech Republic; 5 Leibniz Institute DSMZ-German Collection of Microorganisms and Cell Cultures 28351 , Braunschweig, Germany; 6 Division of Bacterial Molecular Epidemiology and Infectious Diseases, Institute of Veterinary Bacteriology, Vetsuisse Faculty, University of Bern 54179 , Bern, Switzerland; 7 Clinic of Conservative and Preventive Dentistry, Center for Dental Medicine, University of Zurich 27217 , Zurich, Switzerland; 8 Centre for Epidemiology and Microbiology, National Institute of Public Health, Prague, Czech Republic; Anses, Maisons-Alfort Laboratory for Food Safety, Maisons-Alfort, France

**Keywords:** Gram-positive cocci, methicillin resistance, cephalosporin resistance, conjugation, food safety, cold temperature tolerance, linear plasmid

## Abstract

Macrococci are usually found as commensals on the skin and mucosa of animals and have been isolated from mammal-derived fermented foods; however, they can also act as opportunistic pathogens. Here, we used whole-genome sequencing, comparative genomics, extensive biotyping, MALDI-TOF mass spectrometry, and chemotaxonomy to characterize *Macrococcus* sp. strains isolated from livestock and human-related specimens. Based on the results of polyphasic taxonomy, we propose the species *Macrococcus psychrotolerans* sp. nov. (type strain NRL/St 95/376^T^ = CCM 8659^T^ = DSM 111350^T^) belonging to the *Macrococcus caseolyticus* phylogenetic clade. It grows at 4°C, and the core genome of the isolates contains suspected genes contributing to low-temperature tolerance. Variable genetic elements include prophages, chromosomal islands, a composite staphylococcal cassette chromosome island, and many plasmids that affect the overall genome expansion and adaptation to specific ecological settings of the studied isolates. Large plasmids carrying the methicillin resistance gene *mecB* were identified in *M. psychrotolerans* sp. nov. strains and confirmed as self-transmissible to *Staphylococcus aureus in vitro*. In addition to plasmids with circular topology, a 150-kb-long linear plasmid with 14.1-kb-long inverted terminal repeats, harboring many IS elements and putative genes for a type IV secretion system was revealed. The described strains were isolated from human clinical material, food-producing animals, meat, and a wooden cheese board and have the potential to proliferate at refrigerator temperatures. Their presence in the food chain and human infections indicates that attention needs to be paid to this potential novel opportunistic pathogen.

**IMPORTANCE:**

The study offers insights into the phenotypic and genomic features of a novel species of the genus *Macrococcus* that occurs in livestock, food, and humans. The large number of diverse mobile genetic elements contributes to the adaptation of macrococci to various environments. The ability of the described microorganisms to grow at refrigerator temperatures, enabled by genes that are predicted to contribute to low-temperature tolerance, raises food safety concerns. Confirmed *in vitro* conjugative transfer of plasmid-borne *mecB* gene to *S. aureus* poses a significant risk of spread of broad β-lactam resistance. In addition, the intergeneric plasmid transfer to *S. aureus* is indicative of horizontal gene transfer events that may be more frequent than generally accepted. Determining a complete sequence and gene content of linear megaplasmid with exceptional topology for the *Staphylococcaceae* family suggests its possible role in shuttling adaptive traits through an exchange of genetic information.

## INTRODUCTION

Macrococci are Gram-positive, coagulase-negative, and catalase-positive cocci and are phylogenetically closely related to the *Mammaliicoccus* genus. Currently, the *Macrococcus* genus comprises 12 species according to the List of Prokaryotic Names with Standing in Nomenclature (LPSN) (1). They are usually found as commensals on the skin and mucosa of mammals, from where they may also contaminate milk and meat. Case reports on infections by *Macrococcus canis* and *Macrococcus caseolyticus* have been noted in lambs (2), bovine mastitis (3), diseased broiler chicken (4), dogs (5, 6), and skin wound infections, or vulvitis and vaginitis in humans (7, 8). A recent study reported high animal mortality rates associated with infections caused by these bacteria (4). *Macrococcus* species possess various putative enzymatic virulence factors, including lipases, nucleases, and proteases (9). These enzymes are responsible for the ability of the bacteria to cause infection and survive in the host. The genus has gained attention in recent years due to acquiring multiple antibiotic resistance mechanisms through mobile genetic elements (MGEs), including the methicillin resistance genes *mecB* and/or *mecD* (3, 10–13). These genes can be located on a staphylococcal cassette chromosome (SCC*mec*) (14); however, the *mecD* gene was mainly detected on resistance islands McRI*_mecD_* (15) and *mecB* was in addition found on plasmids (16–18). The presence of an almost identical *mecB*-containing plasmid in *Macrococcus canis* and *Staphylococcus aureus* indicates that an exchange of genes between both genera may occur (16, 19).

Comparative genomics studies (7, 9, 15) revealed high plasticity of macrococcal genomes, their pathogenic potential, and the importance of MGEs in horizontal gene transfer (HGT) and pathogenesis. In addition to the acquisition of β-lactam resistance genes, macrococci can acquire other resistance genes by HGT, which leads to multidrug-resistant strains (10, 15, 20, 21). The importance of HGT in the evolutionary processes occurring in macrococci was also supported by the description of *Macrococcus armenti* (22), which acquired antimicrobial genetic elements recently (23).

In this study, a polyphasic taxonomic approach using phenotypic and genotypic methods revealed the taxonomic position of six *Macrococcus* sp. isolates originating from livestock, meat, wooden cheese board, and human clinical samples. The objective is also to characterize MGEs involved in HGT that contribute to genomic plasticity in macrococci and can be transferred to other opportunistic bacterial pathogens of the *Staphylococcaceae* family. The results of genome annotation underscore their significance in medicine and food microbiology.

## RESULTS

### Phylogenetic relationship of novel *Macrococcus* sp. isolates

Six *Macrococcus* sp. isolates were collected from specimens of different origins between 1995 and 2021 (Table 1). Methicillin-resistant *Macrococcus* sp. strains 19Msa1099, 19Msa1047, and 19Msa0499 were collected during a previous study focused on the characterization of macrococci from the nasal cavities of calves and pigs in Switzerland (21).

**TABLE 1:** Source of strains of *Macrococcus psychrotolerans* sp. nov.

Strain^*a*^	Collection numbers^*a*^	Source	Year of isolation	Locality
NRL/St 95/376^T^	CCM 8659^T^, DSM 111350^T^	Wooden cheese board	1995	České Budějovice, Czech Republic
NRL/St 13/116	ND	Human, swab from nose	2013	Příbram, Czech Republic
NRL/St 21/332	ND	Human, pus from wound on leg	2021	Prague, Czech Republic
19Msa1099	CCM 9224	Pork meat	2019	Bern, Switzerland
19Msa0499^*b*^	ND	Calf, nasal swab sample	2019	Bern, Switzerland
19Msa1047^*b*^	ND	Calf, nasal swab sample	2019	Bern, Switzerland

^*a*^ NRL/St, National Reference Laboratory for Staphylococci, National Institute of Public Health (Prague); CCM, Czech Collection of Microorganisms; DSM, German Collection of Microorganisms and Cell Cultures; ND, not deposited.

^*b*^ Strains 19Msa1047 and 19Msa0499 were originally published as *M. caseolyticus* (21).

**Fig 1:**
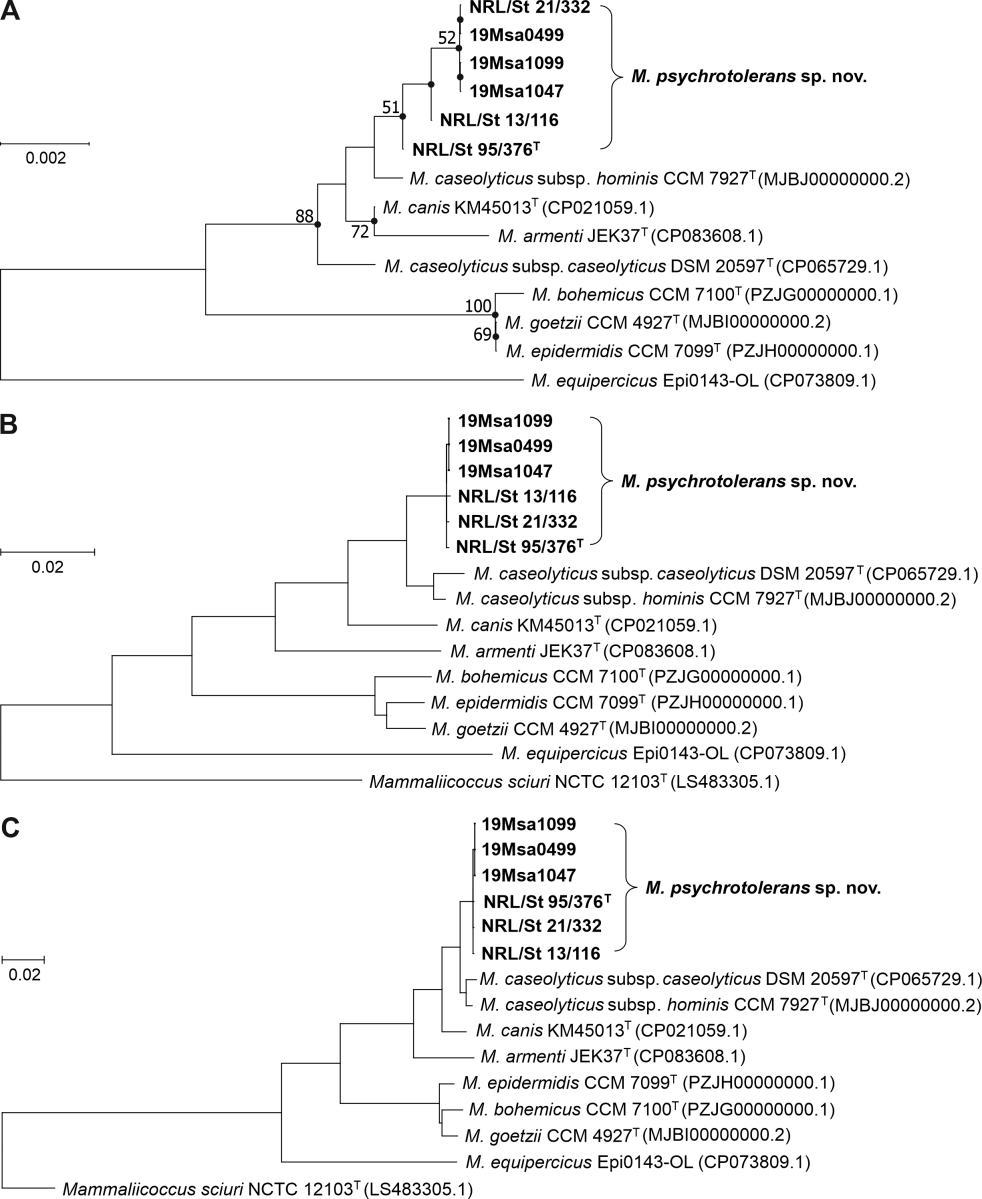
Evolutionary analyses of *Macrococcus caseolyticus* phylogenetic clade, including *M. psychrotolerans* sp. nov. (**A**) Unrooted phylogenetic tree based on complete 16S rRNA gene sequences extracted from whole-genomic sequencing data (GenBank accession numbers of the reference genomes are in parentheses and those of the studied strains in Table S5). The evolutionary history was inferred using the maximum likelihood method and Tamura–Nei model. Filled circles indicate that the corresponding nodes were also obtained in the tree constructed by the neighbor-joining method. The percentage of 500 tree replications above 50% in which the associated taxa clustered together is shown next to the branches. The tree is drawn to scale, with branch lengths measured in the number of substitutions per site. (**B**) Nucleotide sequence-based phylogenetic tree of concatenated alignment of 92 core genes constructed using up-to-date bacterial core gene (UBCG) set. (**C**) Protein sequence-based phylogenetic tree of concatenated alignment of 92 core genes constructed using UBCG. The maximum likelihood tree was inferred using RAxML software and set to 100 replicates.

The phylogenetic analysis of the investigated strains based on complete 16S rRNA gene sequences demonstrated that they belong to the *M. caseolyticus* phylogenetic clade (Fig. 1A). The pairwise nucleotide sequence alignment of the 16S rRNA gene of the most genomically distant isolates NRL/St 95/376^T^ and 19Msa1099 exhibited 99.8% similarity. The NRL/St 95/376^T^ strain, representing the investigated group, exhibited 99.6% pairwise nucleotide similarity with the *M. caseolyticus* subsp. *caseolyticus* DSM 20597^T^ type strain. More precise taxonomic classification of the analyzed strains was determined based on their phylogenetic divergence at the whole-genome level. The calculated average nucleotide identity (ANI) values < 95.5% and digital DNA-DNA hybridization (dDDH) values < 63.5% distinguished all six strains from their closest relatives *M. caseolyticus*, *M. canis*, and *M. armenti* (Table S1), as also shown by protein-coding core genome analysis at the nucleotide and protein level (Fig. 1B and C). The ANI and dDDH values of all six strains were below the threshold values for species delimitation, which are 95%–96% and 70%, respectively (24).

### Discrimination of *Macrococcus* spp. by MALDI-TOF MS and DNA fingerprinting techniques

**Fig 2:**
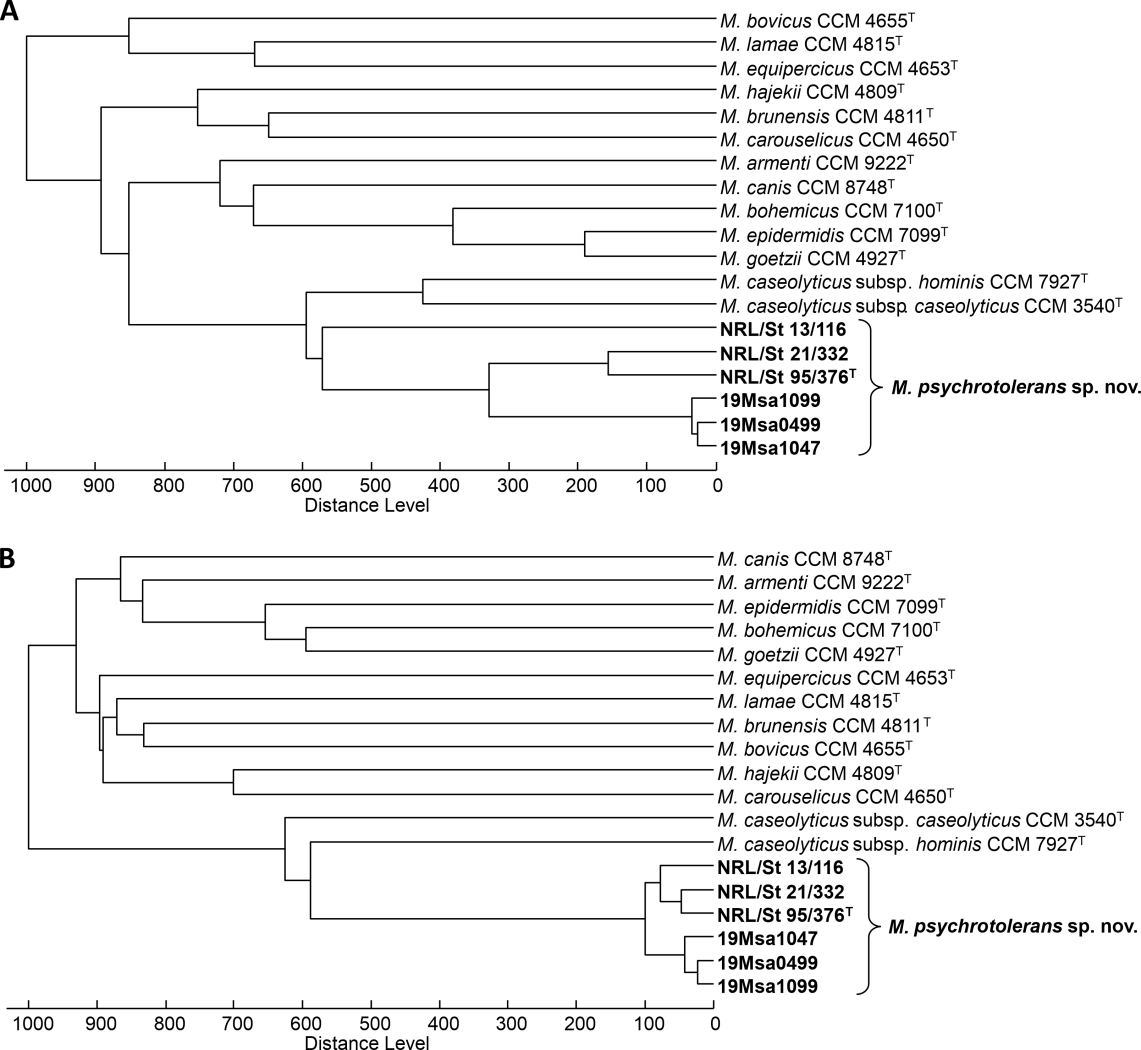
Dendrograms based on MALDI-TOF MS profiles of *Macrococcus psychrotolerans* sp. nov. strains and other members of the genus *Macrococcus* (**A**) using α-Cyano-4-hydroxycinnamic acid (CHCA) as a standard MALDI matrix; (**B**) using alternative MALDI matrix—strongly acidified ferulic acid. The dendrograms were generated using the correlation distance measure with the average linkage algorithm (UPGMA).

Using cluster analysis of matrix-assisted laser desorption/ionization-time-of-flight mass spectrometry (MALDI-TOF MS) data, five of the six novel isolates were separated into a contiguous cluster distinct from phylogenetically related *Macrococcus* spp. (Fig. 2A). The exception to this pattern was strain NRL/St 13/116, grouped more distantly from other isolates. This distinction can be attributed to the close similarity of mass spectra to those of the related *M. caseolyticus* species (Fig. 2A). Therefore, under routine MALDI-TOF MS analysis conditions, strains of *M. psychrotolerans* sp. nov. may not be assigned to the correct *Macrococcus* species. However, when an alternative MALDI matrix (25) that enables the ionization of proteins over a broader range of molecular weights was employed, the novel isolates were grouped into a coherent dendrogram subbranch due to the detection of more species-specific signals (Fig. 2B), which is more accurate for identifying macrococci.

**Fig 3:**
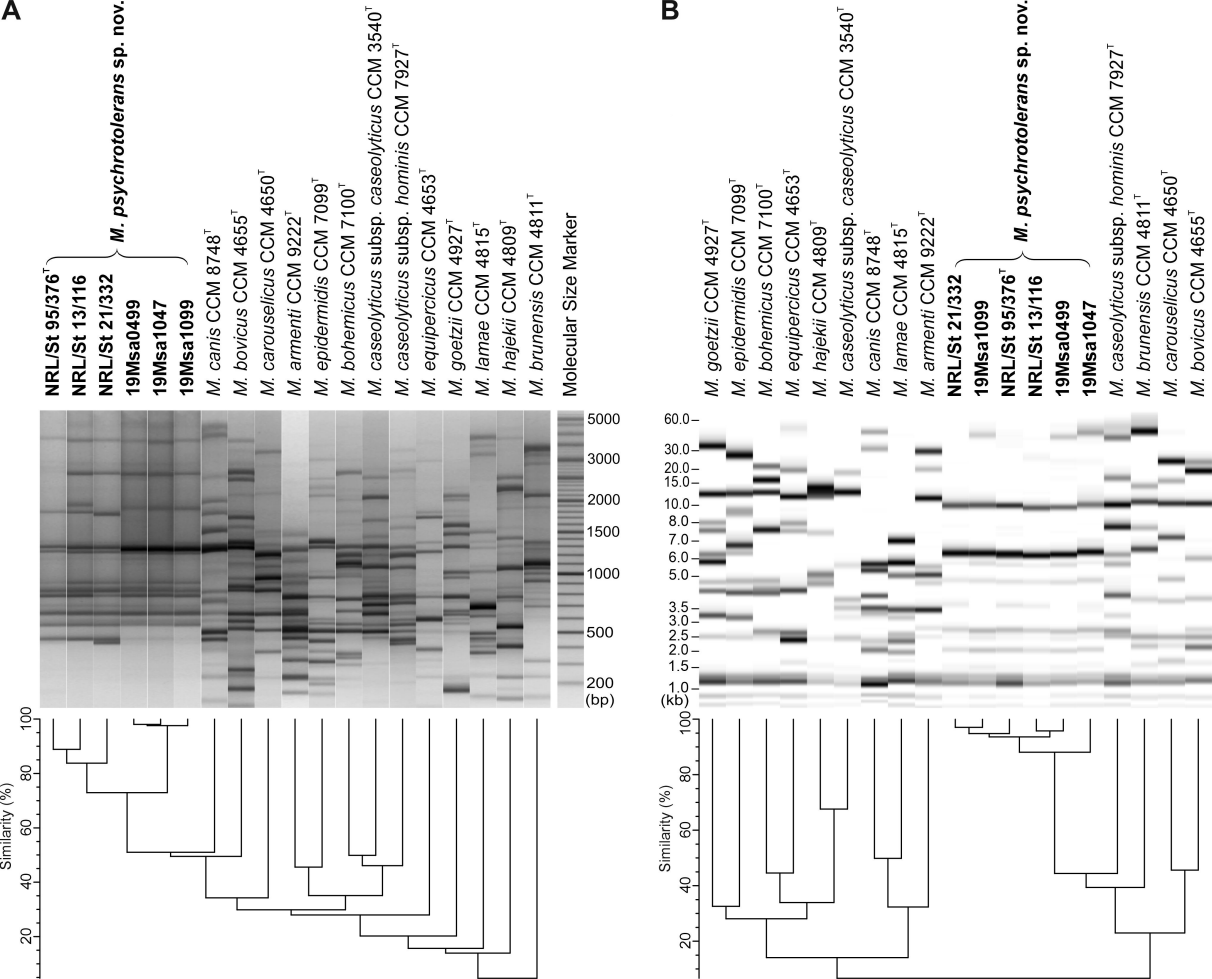
Cluster analysis of DNA fingerprinting data from *Macrococcus psychrotolerans* sp. nov. strains and the type strains of validly named *Macrococcus* spp. (**A**) Dendrogram based on cluster analysis of rep-PCR fingerprints obtained with the (GTG)_5_ primer. (**B**) Dendrogram based on cluster analysis of *Eco*RI ribotype patterns obtained with automated ribotyping using the RiboPrinter system. Both dendrograms were calculated with Pearson’s correlation coefficients with the UPGMA clustering method.

Numerical analysis of the repetitive sequence-based PCR (rep-PCR) fingerprints obtained with the (GTG)_5_ primer (Fig. 3A) and the ribotype profiles obtained by automated ribotyping with the restriction enzyme *Eco*RI (Fig. 3B) grouped all six strains analyzed into a single cluster that was clearly distinct from the fingerprints of the type strains of the validly named *Macrococcus* species. Minor differences due to the presence or absence of some DNA bands allowed the differentiation of certain strains at the strain level; nevertheless, their fingerprints shown by both techniques were visually very similar or identical for strains 19Msa1099, 19Msa1047, and 19Msa0499. These results suggest that both methods are more suitable for the identification of *M. psychrotolerans* sp. nov. than for strain typing.

### Phenotype and physiology

**Fig 4:**
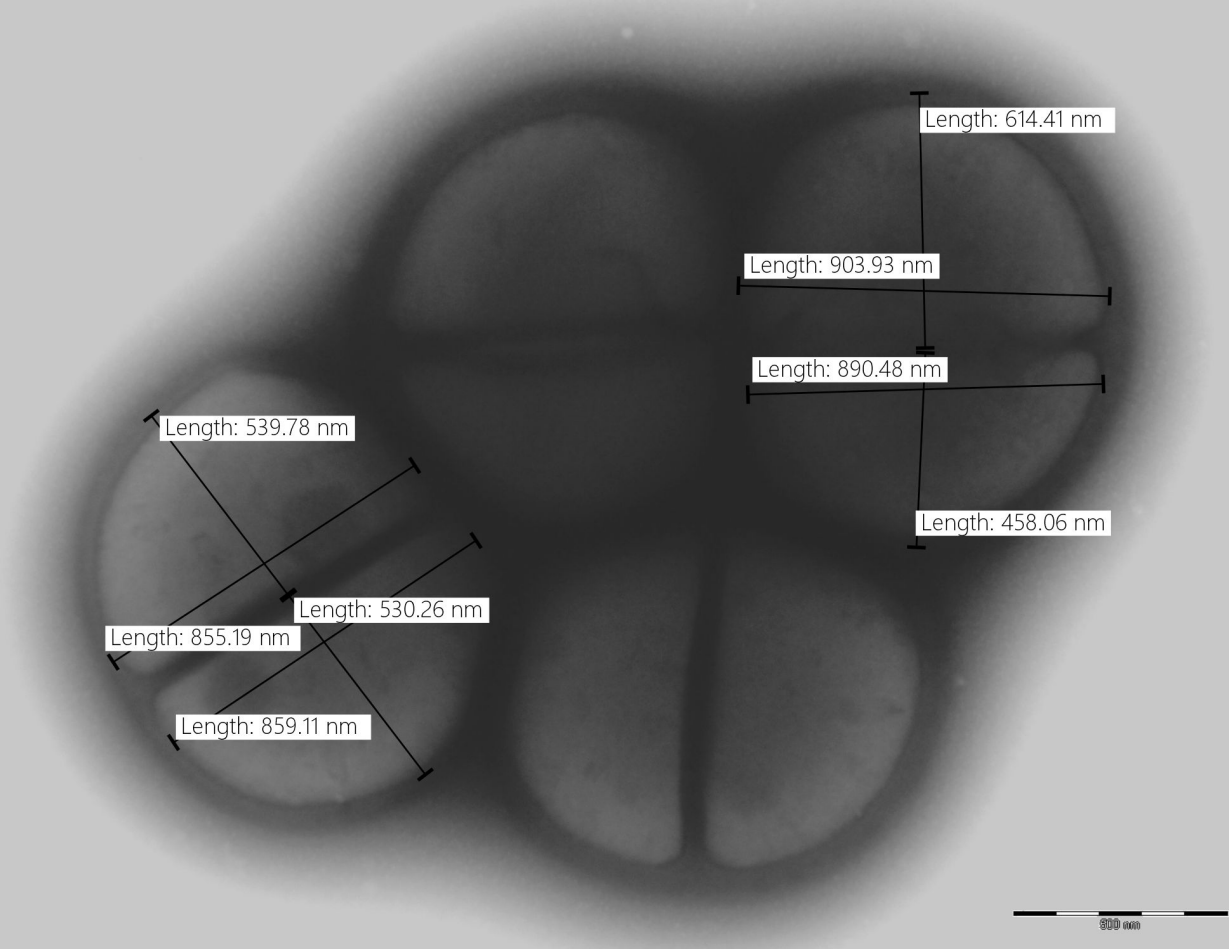
Transmission electron microscopy image of type strain *Macrococcus psychrotolerans* sp. nov. NRL/St 95/376^T^, performed with Morgagni 268D Philips electron microscope. Negative staining was performed using 2% ammonium molybdate. Bar represents 500 nm.

All six investigated strains exhibited typical phenotypic traits corresponding to the basic characteristics of the genus *Macrococcus*. They grew well on standard media used for the routine cultivation of macrococci, e.g., plate count agar (PCA), tryptone soya agar (TSA), nutrient agar (NA), mannitol salt agar (MSA), brain heart infusion agar (BHI), and P agar. All six isolates formed Gram-stain-positive spherical and non-motile cocci. Cells of 890 ± 75 × 610 ± 95 nm occur in pairs or clusters with adjacent sides flattened (Fig. 4). Abundant growth was obtained at a wide range of temperatures (1–45°C) in tryptone soy broth (TSB) and salinities (0%–10% NaCl). All six isolates grew between pH 6.0 and 9.0.

The strains were catalase and oxidase-positive, resistant to bacitracin and susceptible to furazolidone. Complete results of extensive biochemical and physiological testing of the six investigated strains using the conventional tests and the commercial kits API ZYM, API 50CH, and Biolog GEN III MicroPlate test panels are given in the Protologue below. The strain-dependent test results found in the analyzed group are shown in Table S2.

Antimicrobial susceptibility testing using the disc diffusion method showed that all six strains are susceptible to erythromycin, fusidic acid, gentamicin, chloramphenicol, rifampicin, trimethoprim, trimethoprim/sulfamethoxazole, tetracycline, tigecycline, and tobramycin and resistant to penicillin G. Antibiotic susceptibility was strain dependent when tested with ampicillin, oxacillin, cefoxitin, ceftobiprole, ceftaroline, ciprofloxacin, norfloxacin, levofloxacin, clindamycin, linezolid, and novobiocin (Table S3).

Extensive biotyping showed, in agreement with the results of whole-genome phylogeny, DNA fingerprinting techniques, and MALDI-TOF MS data, that the investigated group of six isolates represents a new species of the genus *Macrococcus*. Notably, the ability of the analyzed strains to grow at 1°C in broth seems to be a unique phenotypic feature among the members of *Macrococcus*, which gave the name to the proposed species *Macrococcus psychrotolerans* sp. nov. Physiological and biochemical tests enabling the differentiation of *M. psychrotolerans* sp. nov. from the phylogenetically closely related *Macrococcus* spp. are shown in Table 2.

**TABLE 2:** Differentiation of *Macrococcus psychrotolerans* sp. nov. from closely related macrococci

Test^*a*^	*M. psychrotolerans* sp. nov.^*b*^	*M. canis^b^*	*M. caseolyticus* subsp. *caseolyticus^b^*	*M. caseolyticus* subsp. *hominis^b^*	*M. armenti^b^*
Growth at 1°C^*c*^	+/+	−/n.a.	−/n.a.	−/−	−/n.a.
Growth on mannitol salt agar	+/+	+/+	+/+	+/+	−/−
Voges-Proskauer test	−/d	+/+	+/+	+/+	−/−
Nitrate reduction	+/d	+/+	+/+	+/+	+/+
Pyrrolidonyl arylamidase	+/+	+/+	−/d	+/+	+/+
Acid from^*d*^:					
Glycerol	+/w	w/w	+/+	+/+	−/−
Ribose	+/+	−/−	+/+	+/+	+/+
Galactose	−/−	−/−	+/+	−/−	+/d
Mannitol	−/−	+/+	−/−	+/+	−/d
N-acetyl glucosamine	−/−	−/−	−/−	−/−	w/w
Lactose	−/−	−/−	+/+	−/−	+/d
Yellowish pigment	−/d	−/−	−/−	+/+	−/−

^*a*^ +, positive; −, negative; w, weak reaction; d, 11%–89% strains positive; n.a., not assessed.

^*b*^ Result of type strain/result from species description (5, 7, 22). Data for *M. psychrotolerans* sp. nov. and the type strains of closely related *Macrococcus* spp. were taken from this study in two replications.

^*c*^ Growth in TSB after 7 days.

^*d*^ API 50CH test (bioMérieux).

Cellular fatty acids were analyzed at two temperatures (37°C and 15°C) to understand the role of fatty acids in adaptation to lower temperatures. All six strains resemble a typical fatty acid profile of *Macrococcus* spp. at 37°C (Table 3, complete data in Table S4). Unsaturated fatty acids were enriched at low temperatures on average almost 2× in all strains. There was no statistically significant difference between the isolates of the Czech Republic and Switzerland, but in tendency (at 37°C), Swiss isolates showed a higher abundance of saturated fatty acids compared to the Czech isolates.

**TABLE 3:** Abundance (% of total fatty acids) of pairs of major saturated and unsaturated fatty acids of *Macrococcus psychrotolerans* sp. nov. isolates cultivated at two different temperatures

Fatty acid	Results obtained for tested strains and temperatures											
	NRL/St 95/376^T^		NRL/St 13/116		NRL/St 21/332		19Msa1099		19Msa0499		19Msa1047	
	15°C	37°C	15°C	37°C	15°C	37°C	15°C	37°C	15°C	37°C	15°C	37°C
C_14:1_ ω9c	8.9	1.7	8.6	2.0	7.7	3.1	9.3	1.2	8.9	0.8	8.1	0.7
C_14:0_	19.6	30.5	18.3	31.8	19.7	30.2	20.9	34.0	21.3	39.0	23.8	38.3
C_16:1_ ω11c	26.0	14.4	26.5	16.9	28.37	17.6	29.8	16.2	30.2	14.4	30.0	13.1
C_16:0_	2.4	13.0	2.4	11.8	2.6	11.1	2.3	14.0	2.5	19.7	3.3	20.7
C_18:1_ ω13c	31.6	22.8	30.1	17.7	30.9	21.7	28.7	18.5	27.5	10.9	26.2	10.7
C_18:0_	1.0	6.7	1.0	4.2	1.0	4.5	0.7	6.3	0.8	7.7	1.1	8.8
C_20:1_ ω15c	3.0	1.0	2.5	0.6	2.3	1.3	1.8	0.5	1.7	0.2	1.4	nd^*a*^
C_20:0_	0.9	1.1	0.6	0.6	0.7	0.7	0.3	0.7	0.4	0.4	0.6	0.4
Saturated	26.3	54.5	25.9	56.2	26.3	50.9	26.3	58.8	27.1	69.8	30.5	71.7
Unsaturated	70.7	40.9	68.8	39.0	70.7	45.3	70.6	37.0	70.1	26.7	66.7	24.8

^*a*^ nd, not detected.

### Genomic characterization of *M. psychrotolerans* sp. nov.

**Fig 5:**
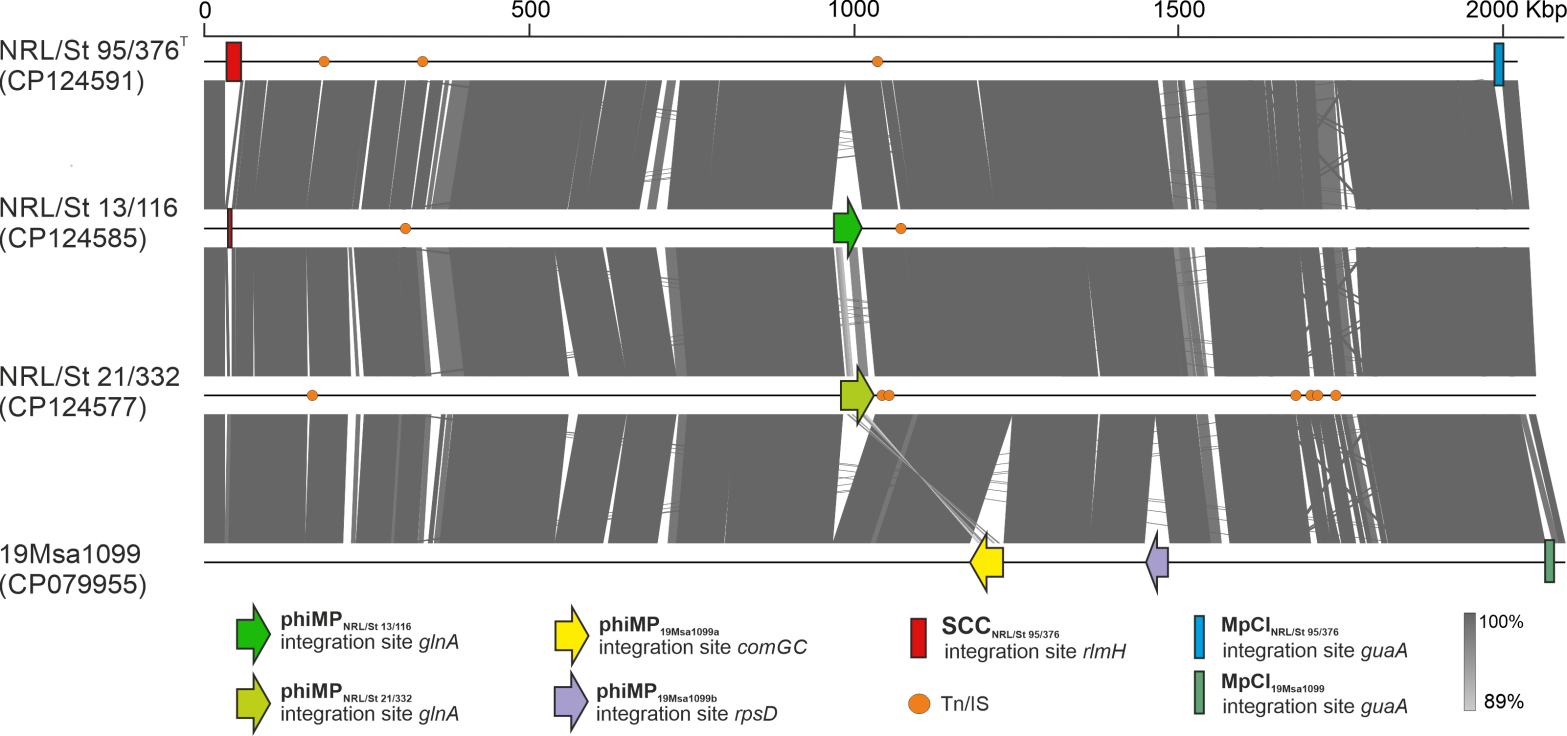
Genome comparison of selected *Macrococcus psychrotolerans* sp. nov. isolates. MGEs, i.e., prophages (phi), SCC, *M. psychrotolerans* chromosomal islands (MpCI), and transposons or insertion sequences are shown and color-coded as in the legend. Chromosomes of strains 19Msa1099, 19Msa0499, and 19Msa1047 share >99% identity and 19Msa1099 is shown as representative.

The *M. psychrotolerans* sp. nov. genomes have a chromosome size of 2.04–2.10 Mb with G+C content of 37.0%–37.2% and contain 2102–2432 predicted protein-coding genes and 79 RNAs (Table S5). They exhibited high similarity, differing primarily in the content of variable genomic elements (Fig. 5). The chromosomes of strains 19Msa1047, 19Msa1099, and 19Msa0499 were almost identical, as also indicated by dDDH and ANI values (Table S1).

The pan-genome of all six *M. psychrotolerans* sp. nov. representatives and the phylogenetically closest *M. caseolyticus* subsp. *caseolyticus* CCM 3540^T^, *M. caseolyticus* subsp. *hominis* CCM 7927^T^, and *M. canis* KM45013^T^ strains share 1659 orthologous clusters of encoded proteins. Thirty-seven distinctive orthologous protein clusters were identified as typical for six *M. psychrotolerans* sp. nov. genomes (Table S6). When we focused on protein coding sequences that were previously not identified in any of the macrococcal genomes available in GenBank from known macrococcal species, 23 unique chromosomal core genes and 14 plasmid-borne genes shared by all isolates were identified for the *M. psychrotolerans* sp. nov. genomes (Table S6). The unique chromosomal genes are often hypothetical or encode integral membrane proteins responsible for the ATP-powered translocation of many substrates across membranes. Distinctive plasmid genes are related to the toxin-antitoxin system essential for maintaining plasmids in a population of cells.

A detailed comparison of representative core genome sequences of *M. psychrotolerans* sp. nov. with closely related macrococci revealed several unique genes with suspected roles in psychrotolerance. One of the distinct operons, consisting of seven genes, encodes proteins involved in acetoin catabolism (dihydrolipoyl dehydrogenase, thiamine pyrophosphate-dependent dehydrogenase, alpha-ketoacid dehydrogenase, dihydrolipoamide acetyltransferase, and dihydrolipoamide acetyltransferase) (Table S6). According to the Gene Ontology (GO) enrichment analysis (26) among strains belonging to *M. psychrotolerans* sp. nov., these genes belong to an enrichment pathway related to glycolytic processes and/or to the acetoin metabolism process, which involves the breakdown of carbohydrates into pyruvate, accompanied by the production of metabolites with a possible role in the psychrotolerance of these microorganisms (27).

In addition, all the *M. psychrotolerans* sp. nov. strains encode three different putative cold shock proteins, also identified sporadically in genomes classified in GenBank as *M. canis*, *M. caseolyticus,* or *M. armenti*, where the reciprocal amino acid identity ranges from 96.9% to 93.9% (query coverage 98%). The cold shock protein annotated as CspA (GenBank: WZE69993) shares 62.5% amino acid identity (94% query coverage) with the cold shock protein CspA (GenBank:VWQ03685) of *Escherichia coli*. Two other cold shock proteins (GenBank: WZE71216 and WZE71217) belonging to the CspC protein superfamily (UniProt: P0A9Y6) are genes located upstream that belong to the putative acetoin catabolism operon and separated by three genes from this operon.

### Chromosomal MGEs

**Fig 6:**
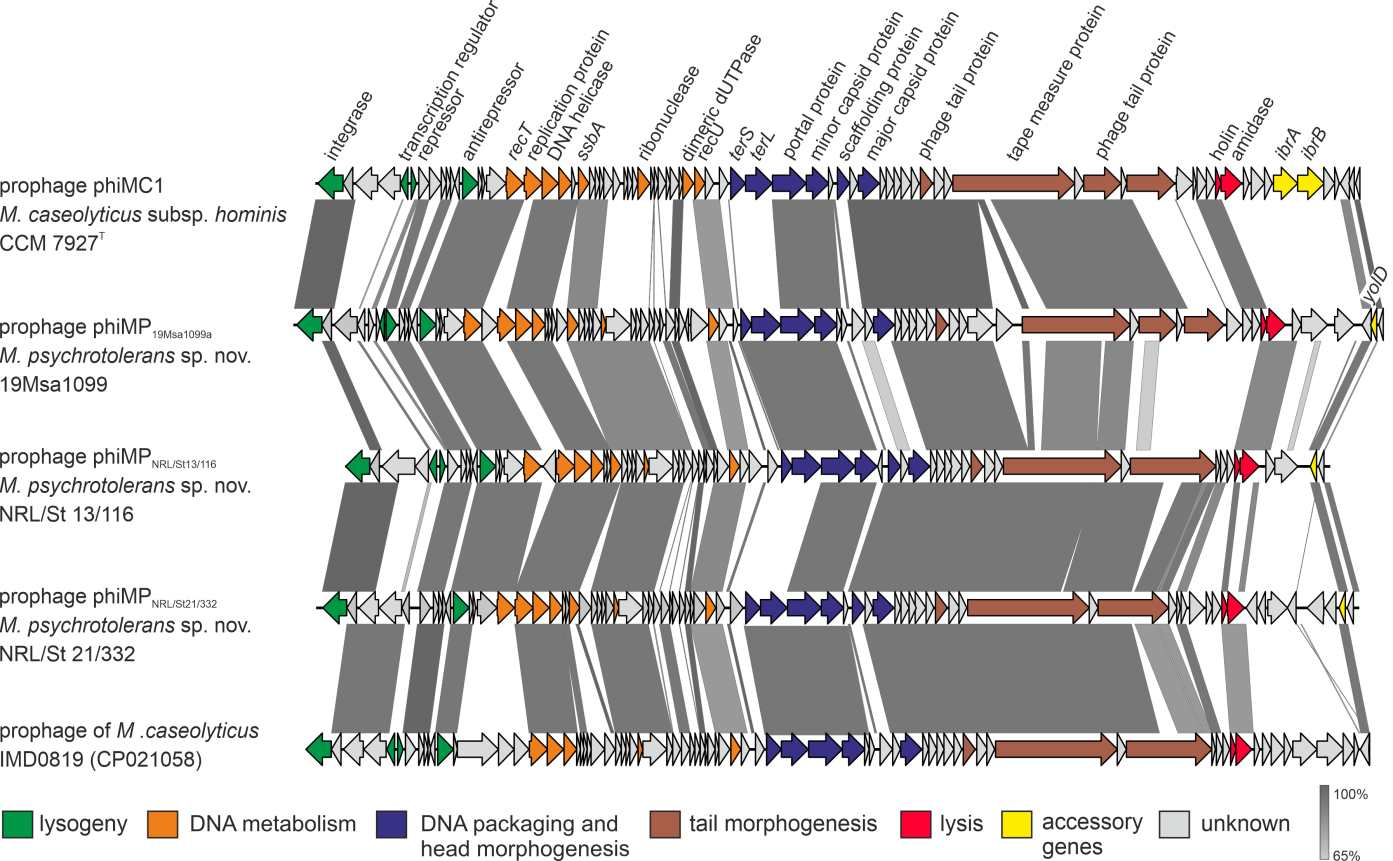
Genome comparison of bacteriophages identified in *Macrococcus psychrotolerans* sp. nov. with closely related bacteriophages from *Macrococcus caseolyticus*. Genomes were aligned using the blastn algorithm, and similar regions with more than 65% identity are indicated. Arrows represent the positions and orientations of the coding regions. Predicted genome modules are color-coded according to the legend.

The *M. psychrotolerans* sp. nov. strains have up to two complete prophages in their genomes integrated into three different *att* sites (Fig. 5). The prophage genomes exhibit low similarities to any known viruses from the order *Caudovirales* according to NCBI entries. They are likely yet undescribed phage genera. Prophages have a modular structure typical for staphylococcal siphoviruses (28) but a distinct gene composition. Comparative analysis showed that the prophages designated phiMP_NRL/St 13/116_ (47.2 kb), phiMP_NRL/St 21/332_ (49.3 kb), and phiMP_19Msa1099a_ (52.3 kb) are highly similar in their major genomic modules to the previously described phage ϕMC1 (7) from *M. caseolyticus* subsp. *hominis* strain CCM 7927^T^ (coverage 57% and nucleotide identity 96.5%) and with the prophage identified in *M. caseolyticus* strain IMD0819 (3) (coverage 62% and identity 91.5%) but differ in their integrase genes (Fig. 6).

The prophages phiMP_NRL/St13/116_ and phiMP_NRL/St21/332_ share 95.7% mutual identity and are integrated into bacterial genomes downstream of the *glnA* gene encoding type I glutamate-ammonia ligase (GenBank: QYA77299.1). Two prophages were identified in strains 19Msa1099, 19Msa0499, and 19Msa1047. The first, designated phiMP_19Msa1099a_, is integrated into the *comGC* gene, a part of the *comG* operon involved in binding of transforming DNA, which is, thus, disrupted by a negative lysogenic conversion process. The same integration site has been described previously in phage ϕMC1 (7). The second 38.6 kb-long prophage designated phiMP_19Msa1099b_ is integrated into the *rpsD* gene encoding 30S ribosomal protein S4 (WP_219492567.1).

**Fig 7:**
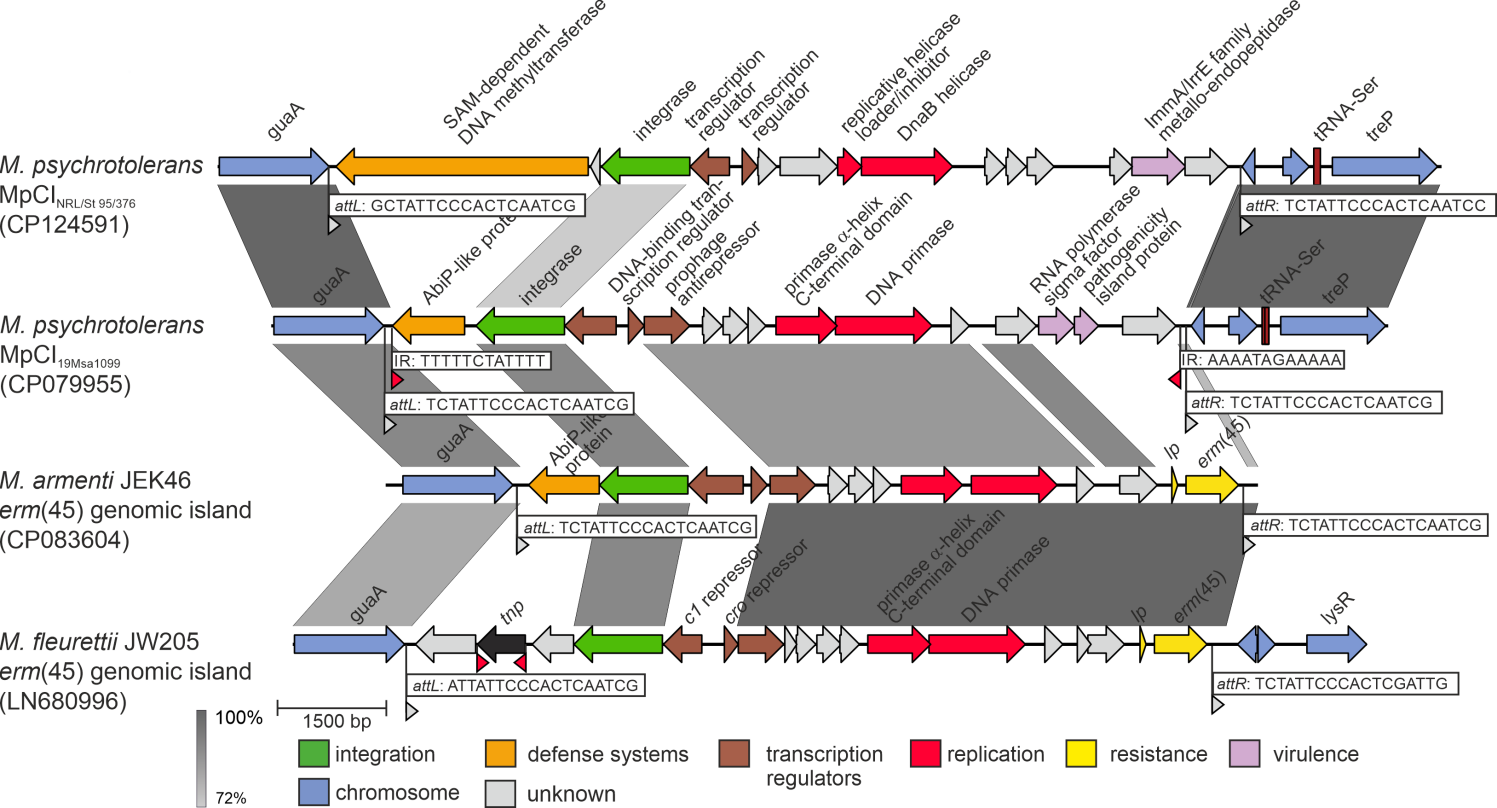
Global alignment of phage-inducible chromosomal islands identified in *Macrococcus psychrotolerans* sp. nov. strains NRL/St 95/376^T^ and 19Msa1099. MpCI_19Msa1099_ is identical to islands in 19Msa1047 and 19Msa0499. The GenBank accession numbers of whole genomic sequences are provided.

Comparative genomic analyses revealed the presence of 12.7 kb chromosomal island (CI) in strain NRL/St 95/376^T^ (designated MpCI_NRL/St 95/376_) and 11.2 kb CI in strains 19Msa1099, 19Msa1047, and 19Msa0499 (designated MpCI_19Msa1099_) at the same integration site between the tRNA_Ser_ gene and the guanosine monophosphate (GMP) synthetase gene *guaA* (GenBank: WZE70899.1) (Fig. 7). These CI belong based on conserved modular structure, with genes clustered according to function, into phage inducible chromosomal islands group (29). A putative site-specific integrase of MpCI_NRL/St 95/376_ exhibits 76.5% amino acid identity the MpCI_19Msa1099_ integrase. In other *Macrococcus* spp. genomes except for *M. armenti* (23), this CI is absent or is replaced by a gene for putative SdrD B-like domain-containing protein (GenBank: WP_219503173.1), which is similar to the SpaA isopeptide-forming pilin-related protein (GenBank: WP_146606923.1). A structurally similar genomic island integrated into the *guaA* gene has been reported in *Mammaliicoccus fleurettii* along with the gene *erm*(45) for macrolides-lincosamides-streptogramin B (MLS_B_) resistance (23, 30) (Fig. 7). MpCI_NRL/St 95/376_ encodes a putative SAM-dependent type IIS restriction-modification enzyme (GenBank: WP_101142918), suggesting a possible protective function of this element from the host immune system. MpCI_19Msa1099_ carries a gene for an Abi-like family protein (GenBank: WP_219493404) similar to the *Lactococcu*s sp. abortive infection protein, which is involved in bacteriophage resistance (31).

*Macrococcus psychrotolerans* sp. nov. strain NRL/St 95/376^T^ carries a 24.86-kb-long composite island designated SCC_NRL/St 95/376_ that belongs to SCC elements with *ccrB_m2_A_m2_* recombinase gene complex. The SCC_NRL/St 95/376_ element is bordered by an almost identical 28 bp direct repeat (TGAATCGTATCAYAAGTAATGAGGTTTA) and inserted into the *rlmH* gene. It has 23 putative CDS. The *ccr* gene complex shares homology with *ccr* genes of *M. canis* KM45013 SCC*mec* (32) and *M. caseolyticus* JCSC7096 (18). Apart from the conserved *ccr* gene complex, the cassette harbors a DNA methyltransferase gene and a cluster of genes with unknown functions.

### Multiple plasmid carriage, including a linear megaplasmid in *M. psychrotolerans* sp. nov.

Multiple plasmids are present in all six investigated *M. psychrotolerans* sp. nov. strains, ranging from 5 to 11 plasmid replicons. Plasmids smaller than 10 kb contained predominantly *repUS56* and *rep7a* genes (Table S7), which are the dominant replication genes in plasmids belonging to the *M. caseolyticus* phylogenetic group, with a prevalence of 35% and 34%, respectively, and are frequently associated with antimicrobial resistance (20). The identified plasmid replicons also frequently contain genes for both mobilization and toxin-antitoxin systems (Table S7).

Large 66.4-kb to 68.6-kb *mecB* gene-carrying plasmids were identified in three methicillin-resistant *M. psychrotolerans* sp. nov. strains 19Msa1099, 19Msa0499, and 19Msa1047 (Table S7) (21). The presence of the *mecB*-encoding plasmid is consistent with the phenotypic resistance of these three strains to oxacillin and cefoxitin (Table S3). These plasmids also harbor genes with homology to the type IV conjugative DNA transfer secretion system (GenBank: QYA38970.1), which seems to be widely used as a means for transferring plasmids in Gram-positive bacteria (33, 34).

**Fig 8:**
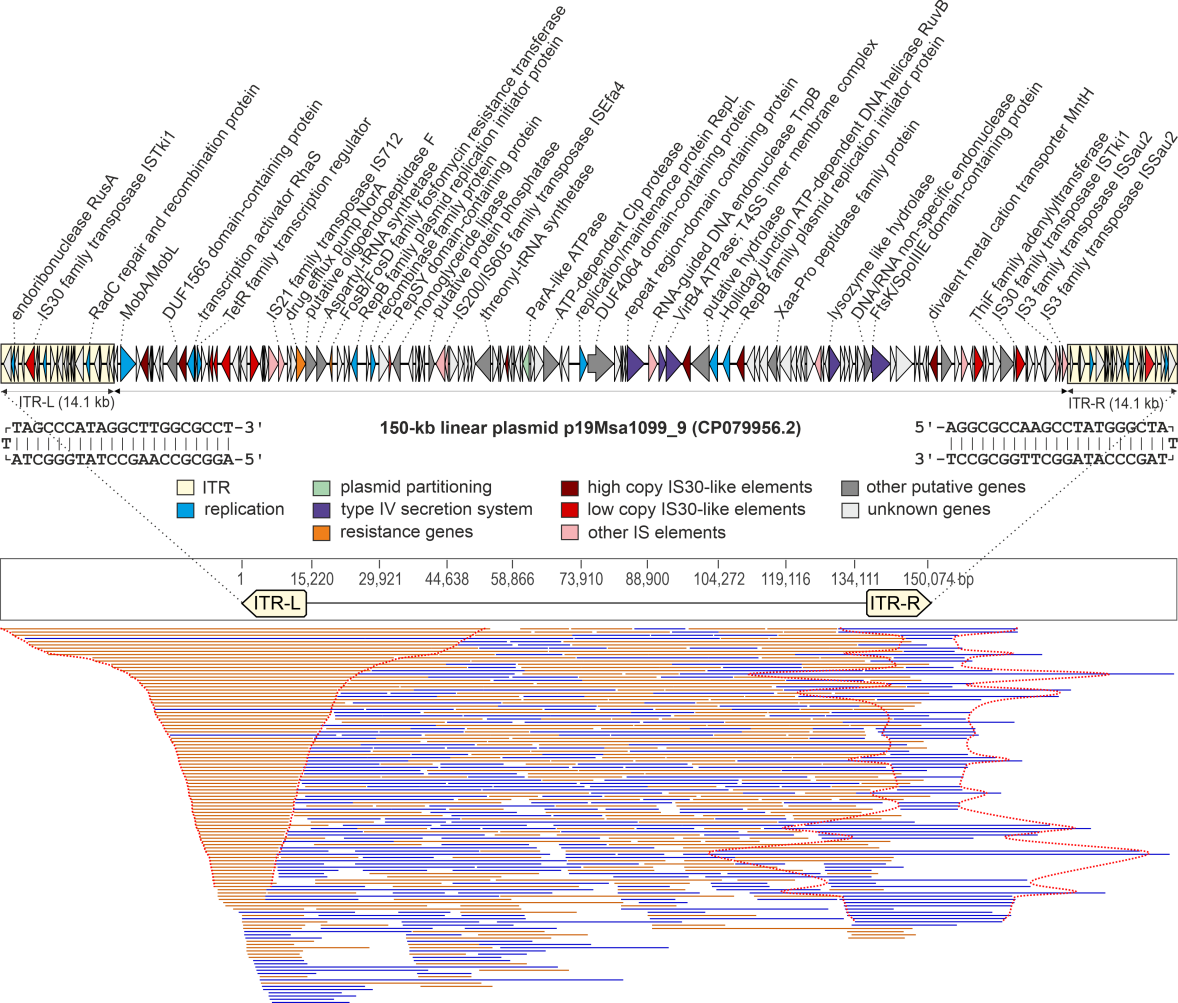
Genetic map of linear plasmid p19Msa1099_9 (GenBank accession number CP079956.2). Arrows show the position and orientation of predicted coding regions. The predicted genes are color-coded according to the legend. The sequences of physical ends of the hairpin plasmid are shown below the gene map. The mapping of sequencing reads longer than 10 kb to the assembled plasmid sequence revealed a centrally symmetric alignment of reads to the covalently closed ends of the hairpin plasmid. These reads resulted from the unfolding of the terminal hairpin structure during Oxford Nanopore sequencing technology (ONT) as it passed through the nanopore, and therefore, the unmapped parts correspond to the complementary strand (highlighted by a red dotted line). The mean coverage calculated from all ONT reads by Geneious mapper was 881× with a standard deviation of 215.

In addition to the circular plasmids, almost identical 150 kb-long megaplasmids p19Msa1099_9, p19Msa0499_10, and p19Msa1047_11 with a covalently closed linear topology have been identified in the three strains 19Msa1099, 19Msa0499, and 19Msa1047. The plasmids have 14.1-kb-long inverted terminal repeats (ITRs) terminated by a covalent closure site apparent from centrally symmetric long sequencing reads originating from double-stranded plasmid ends (Fig. 8). The first and last T bases have no complementary counterparts and link both DNA chains (Fig. 8). The gene homologs for the RadC protein and the endoribonuclease RusA, which are involved in DNA repair and homologous recombination, were annotated inside ITRs. The linear plasmid also encodes two distinct RepB family plasmid replication initiator proteins, proteins responsible for mobilization (MobA/MobL), cell division (FtsK/SpoIIIE domain-containing protein), and plasmid segregation (ParA-like ATPase). There are several predicted genes in the plasmid sequence that encode protein domains found in type IV secretion systems (T4SS) (Fig. 8). The gene homolog for the multidrug efflux pump NorA (Fig. 8), which can confer resistance to fluoroquinolones and other structurally unrelated drugs, was identified in the plasmid (35). In addition to these genes, there are a number of IS elements and genes with unknown functions (Fig. 8).

### *In vitro mecB*-mediated β-lactam antibiotic resistance transfer to *Staphylococcus aureus* by conjugation

The ability of 66.4 kb-long *mecB*-harboring plasmid p19Msa1099_1 (GenBank: CP079957) to transfer from *M. psychrotolerans* sp. nov. 19Msa1099 into the laboratory strain *S. aureus* RN4220 (pT181) was demonstrated. Together with p19Msa1099_1, the transferred 1.9 kb cryptic plasmid p19Msa1099_8 was detected in the *S. aureus* transconjugants. Analysis of sequence reads of the obtained transconjugants showed a mean coverage of 574.1 for the RN4220 genome, 1,244.2 for plasmid pT181, 23.1 for plasmid p19Msa1099_1, and 238.8 for plasmid 19Msa1099_8. There was no evidence of sequence reads mapping to the chromosome of the donor strain 19Msa1099. The stability of the *mecB* gene-carrying plasmid in the *S. aureus* RN4220 (pT181, p19Msa1099_1, p19Msa1099_8) cells during replication is 80% when the cultures were grown for 24 hours on plates without antibiotic selection. The resistance phenotype of tested penicillins and cephalosporins of the transconjugant *S. aureus* after acquiring the *mecB* gene-carrying plasmid was almost the same as the donor *M. psychrotolerans* sp. nov. 19Msa1099. Ceftobiprole resistance and decreased ceftaroline susceptibility resulted from acquiring the *mecB* gene in the *S. aureus* transconjugant (Table S3).

### Taxonomic description of *M. psychrotolerans* sp. nov.

*Macrococcus psychrotolerans* sp. nov. (psy.chro.to´ler.ans. Gr. masc. adj. *psychros* cold; L. pres. part. *tolerans* tolerating; N.L. part. adj. *psychrotolerans* cold-tolerating).

Cells are Gram-stain positive spherical cocci with variable size of 890 ± 75 × 610 ± 95 nm, occurring predominantly in pairs, or tetrads and clusters, aerobic, non-spore-forming and non-motile. Colonies on TSA agar are circular, whole margin, flat, smooth, matt to glossy, 2 mm in diameter after 48 h of growth, and predominantly beige. Negative hemolytic activity on Columbia sheep-blood agar. Positive growth on PCA, TSA, NA, MSA, BHI, and P agar. Strains grow in the presence of up to 10% NaCl, at 1°C and 45°C, but not at 50°C, and in the range of pH 6 to pH 9. Catalase, oxidase, and pyrrolidonyl arylamidase positive. Coagulase, arginine dihydrolase, urease, and ornithine decarboxylase negative. Susceptible to furazolidone (100 µg) and resistant to bacitracin (10 IU). Hydrolysis of esculin and Tween 80 negative. Variable reactions were obtained for the hydrolysis of DNA, gelatine and glycerol, nitrate reduction, Voges-Proskauer test, clumping factor, and novobiocin resistance.

Enzymatic reactions tested with the API ZYM kit were positive for alkaline phosphatase, esterase (C4), and esterase lipase (C8), negative for lipase (C14), valine arylamidase, cystine arylamidase, trypsin, α-galactosidase, β-galactosidase, β-glucuronidase, β-glucosidase, N-acetyl-β-glucosaminidase, α-mannosidase, and α-fucosidase and variable for α-glucosidase, α-chymotrypsin, acid phosphatase, leucine arylamidase, and naphthol-AS-Bi-phosphohydrolase.

Acid production from substrates tested with the API 50CH kit was positive for ribose, D-glucose, D-fructose, maltose, and trehalose; negative for erythritol, D-arabinose, L-arabinose, D-xylose, L-xylose, adonitol, β-methyl-D-xyloside, galactose, mannose, sorbose, rhamnose, dulcitol, inositol, mannitol, sorbitol, α-methyl-D-mannoside, α-methyl-D-glucoside, N-acetyl glucosamine, amygdaline, arbutin, salicin, cellobiose, lactose, melibiose, inulin, melezitose, D-raffinose, starch, glycogen, xylitol, β-gentiobiose, D-turanose, D-lyxose, D-tagatose, D-fucose, L-fucose, D-arabitol, L-arabitol, 2 keto-gluconate, and 5 keto-gluconate and variable for glycerol, sucrose, and gluconate.

Phenotype characterization using Biolog GEN III MicroPlate test panels exhibited positive reactions for the utilization of dextrin, D-maltose, D-fructose, glycerol, D-glucose-6-PO_4_, D-fructose-6-PO_4_, L-alanine, L-histidine, L-serine, D-gluconic acid, L-lactic acid, α-keto glutaric acid, acetoacetic acid, α-hydroxy-butyric acid, formic acid, and acetic acid; borderline utilization of inosine; negative reactions for the utilization of D-cellobiose, gentiobiose, sucrose, stachyose, D-raffinose, α-D-lactose, D-melibiose, β-methyl-D-glucoside, D-salicin, N-acetyl-D-glucosamine, N-acetyl-β-D-mannosamine, N-acetyl-D-galactosamine, N-acetyl neuraminic acid, D-mannose, D-galactose, 3-methyl glucose, L-rhamnose, D-sorbitol, D-mannitol, D-arabitol, myo-inositol, D-aspartic acid, D-serine, L-arginine, L-aspartic acid, pectin, D-galacturonic acid, L-galactonic acid lactone, D-glucuronic acid, mucic acid, quinic acid, D-saccharic acid, p-hydroxy phenylacetic acid, D-lactic acid methyl ester, citric acid, D-malic acid, bromo-succinic acid, Tween 40, γ-amino-butyric acid, β-hydroxy-D,L-butyric acid, and propionic acid; and variable reactions for the utilization of D-trehalose, α-D-glucose, D-turanose, D-fucose, L-fucose, gelatine, glycyl-L-proline, L-glutamic acid, L-pyroglutamic acid, glucuronamide, methyl pyruvate, L-malic acid, and α-keto butyric acid. The strains grow in the presence of 1% sodium lactate, nalidixic acid, lithium chloride, potassium tellurite, aztreonam, and sodium butyrate; do not grow in the presence of fusidic acid, troleandomycin, rifamycin SV, minocycline, lincomycin, guanidine HCl, niaproof 4, vancomycin, tetrazolium violet, tetrazolium blue, and sodium bromate, and the growth was variable for D-serine when tested using Biolog GEN III MicroPlate test panels.

The type strain NRL/St 95/376^T^ contains menaquinones MK-6 (99.3%) and MK-7 (0.7%), and the peptidoglycan type is L-Lys-Gly_3-4_ (A11.2, A3α). The predominant fatty acids are C_14:0_ (30.5%), C_18:1_ ω13c (22.8%, identified as C_18:1_ ω9c by the MIDI system), C_16:1_ ω11c (14.4%), C_16:0_ (13.0%), C_18:0_ (6.7%), and C_14:1_ ω9c (1.7%, not identified by the MIDI system). Furthermore, C_16:0_ alcohol (2.3%) and C_18:0_ alcohol [1.9%, identified as C_18:3_ ω6c (6, 9, 12) by the MIDI system] are detectable. The genomic DNA G+C content of the type strain NRL/St 95/376^T^ is 37.2%. Type strain NRL/St 95/376^T^ (= CCM 8659^T^ = DSM 111350^T^) was isolated from a wooden cheese board.

## DISCUSSION

*Macrococcus* species belong to the normal flora of several mammals, and some species may be occasionally associated with animal infections, particularly livestock and pets. Certain species of the genus *Macrococcus* have been found in food products, mainly raw milk and dairy products. Bello et al. (36) suggested the reclassification of certain *Macrococcus* spp. into the novel genus *Macrococcoides* based on conserved signature indels as the main taxonomic criterium without studying other traits. According to LPSN (1), we consider the *Macrococcoides* genus to be a synonym due to nomenclature inaccuracies. Here, we described *M. psychrotolerans* sp. nov., which is closely related to the *M. caseolyticus* phylogenetic group and, like *M. caseolyticus,* includes isolates from animal husbandry and meat and dairy processing environments. *M. caseolyticus* is the most studied species of the *Macrococcus* genus and is used as a starter culture, contributing to the aroma and flavor of fermented foods. However, concerns are arising regarding food safety due to recent findings of methicillin and other antibiotic resistance genes in *M. caseolyticus*. These genes pose a risk to human health, as they can be transferred to other bacteria, particularly staphylococcal species present in food (11). The emergence of human-related isolates suggests that these organisms may sporadically enter the human clinical field.

The genome of *M. psychrotolerans* sp. nov. only differs marginally from related *M. caseolyticus* in G+C genome content, genome size, and median number of proteins encoded by the core genome. In contrast to other macrococci, strains of *M. psychrotolerans* sp. nov. can grow at refrigerator temperatures. The ability of a microorganism to survive and grow at low temperatures can allow the contamination of food such as meat or dairy products, which was noted in a recent metagenomic study in which *Macrococcus* sp. represented the predominant genus next to *Lactococcus* sp. during refrigerated meat storage (37). Comparative analysis of *M. psychrotolerans* sp. nov. genomes revealed the presence of a combination of genes for acetoin metabolism, butanediol dehydrogenase (BDH), ABC transporter genes, and three genes encoding cold shock proteins, which may support the ability of this bacteria to grow at low temperatures. Beckering et al. (27) found that the *Bacillus subtilis* gene cluster encoding the ABC transporter for acetoin was upregulated by a rapid decrease in temperature from 37°C to 15°C. It suggests that metabolic derivatives of acetoin could function as cryoprotectants under cold shock conditions (27). One probable metabolite with a possible cryoprotective function is 2,3-butanediol (38), which is metabolized from acetoin by 2,3-butanediol dehydrogenase (39). It has a low freezing point of −60°C and, in combination with specific proteins, can act as an antifreeze compound (40).

Members of small cold shock proteins are the most common bacterial proteins that react to temperature decreases and have canonical nucleic acid-binding sequence motifs (41). The homolog of CspA identified in *M. psychrotolerans* sp. nov. strains is related to extensively studied CspA (GenBank: WP_000014594) of *E. coli* (62.5% amino acid identity). It protects mRNA secondary structures and, thus, has a fundamental effect on cellular transcription during a rapid decrease in temperature (42). In the other two products of *csp* genes classified into the CspC protein superfamily, canonical nucleic acid-binding sequence motifs RNP1 and RNP2 have been predicted to bind to single-stranded RNA and DNA (41). Both the *csp* genes are localized in tandem, have distinct promoters and terminators, and have a lower G+C content (31.21% on average) than the genome average, indicating their horizontal transfer. Nevertheless, both *csp* gene products exhibit high mutual amino acid identity, differing in only two amino acids. A BLAST search only sporadically found the presence of these genes in sequence records of *M. caseolyticus* and *M. canis* and of the genus *Lysinibacillus* belonging to *Bacillales* (*Caryophanales*). However, the genes had a distinct regulatory sequence for transcription, and no information about the phenotype related to growth at low temperatures is known.

The cellular fatty acid profiles of *M. psychrotolerans* sp. nov. showed a clear overlap with *M. canis* and *M. caseolyticus* but combine features of both related species. The abundance of *iso*-fatty acids is lower than in *M. caseolyticus*, while the fatty alcohol abundance is far lower than in *M. canis* (5). In contrast, the type strain of *M. psychrotolerans* sp. nov. contained higher amounts of the unsaturated C_18:1_ ω13c (identified as C_18:1_ ω9c in the MIDI system) and C_16:0_. The ratio of saturated and unsaturated fatty acids is a potential key feature for temperature adaptation and the regulation of membrane fluidity and might be one of the features relevant for the psychrotolerance of *M. psychrotolerans* sp. nov. Thus, we studied the fatty acid profile at two different temperatures (37°C as for published reference strains and 15°C). In all isolates, we observed the shift from saturated to non-saturated fatty acids by a factor of almost 2, which is a common principle in many species to modify the fluidity of the membrane (43, 44).

All *M. psychrotolerans* sp. nov. genomes contain one or more prophages integrated into different *att* sites. Prophage phiMP_19Msa1099a_, identified in three *M. psychrotolerans* sp. nov. strains 19Msa1099, 19Msa0499, and 19Msa1047, is integrated into the *comGC* gene. The *comG* operon-encoded proteins exhibit similarity to genes for type IV pillar assembly (45) and for the secretion of some proteins in Gram-negative bacteria (46). Its insertional inactivation in *comGC* leads to a loss of transformability and the failure to bind DNA by cells growing in competency state (46, 47). We described this phenomenon previously in *M. caseolyticus* subsp. *hominis*, in which this negative lysogenic conversion led to nearly identical genomes of unrelated strains (7). Therefore, we hypothesize that the insertional inactivation of the *comGC* gene by prophage contributed to the loss of the ability of natural transformation in the three *M. psychrotolerans* sp. nov. strains. This may be the reason for their almost complete identity, despite the fact they originate from different sources, but we cannot exclude the possibility of a clonal relationship.

The variable part of the genomes of strains NRL/St 95/376^T^, 19Msa1099, 19Msa1047, and 19Msa0499 includes CIs integrated into the 3′ end of the GMP synthetase gene (*guaA*), a known integration site for genomic islands, transposons, and bacteriophages in various bacterial species (48, 49). Also, a number of staphylococcal pathogenicity islands (SaPIs) integrate into this site (50). The MpCI_19Msa1099_ identified in 19Msa1099, 19Msa1047, and 19Msa0499 strains exhibits 86.7% nucleotide similarity (57% query coverage) with an island identified in *M. armenti* (23) and 87.4% nucleotide similarity (53% query coverage) with an 11.5 kb genomic island of *M. fleurettii* encoding an MLS_B_ resistance methylase Erm(45) (30), which suggests that there is an intra- and interspecies and intergeneric transmission of whole islands or their parts.

Most *M. psychrotolerans* sp. nov. strains contain a high number of plasmids, including conjugative plasmids larger than 66 kb encoding the *mecB* gene and also linear megaplasmids (Table S7). *M. psychrotolerans* sp. nov. strains were isolated in different regions and may inhabit various habitats. The *mecB* gene-carrying plasmids and linear megaplasmids were only carried by strains 19Msa1099, 19Msa0499, and 19Msa1047 isolated in Switzerland. This indicates variability in antibiotic resistance and may also suggest recent plasmid acquisition from other bacteria.

The *mecB*-encoded penicillin-binding protein 2a (PBP2a)-related transpeptidase was found on large plasmids and SCC*mec* elements in *Macrococcus* spp. (7, 17, 18, 21, 32). The *mecB* gene-encoding plasmids identified in strains 19Msa0499, 19Msa1099, and 19Msa1047 are similar to plasmid pSAWWU4229 (GenBank: LT799381) from *S. aureus* strain UKM4229 obtained from a 67-year-old cardiology patient with no signs of infection (19), to plasmid pKM0218 from *M. canis* (16) and to plasmid pMCCL2 (GenBank: AP009486) from *M. caseolyticus* (17). This pMCCL2 carries the *mec* gene complex (*mecB-mecR1*_m_-*mecI*_m_-*blaZ*_m_) as part of a transposon Tn*6045* (18), which is also carried on plasmids of strains 19Msa0499, 19Msa1099, and 19Msa1047. Similarly, plasmids with high nucleotide similarity were identified in methicillin-resistant *M. caseolyticus* isolated from cattle in Switzerland (21) and England and Wales (10, 15). Evidence of the occurrence of similar plasmids in different locations and isolates suggests that they are transferable *in vivo* or in the environment. *M. psychrotolerans* sp. nov. strains harbor several plasmids that carry genes for mobilization (*mobA*, *mobC*, and *mobL*), suggesting their possible role in horizontal transfer.

Here, we proved *in vitro* conjugative transfer of *mecB* encoding plasmid p19Msa1099_1 from *M. psychrotolerans* sp. nov. to *S. aureus*. This intergeneric transfer poses a risk of spreading resistance to beta-lactams to other human opportunistic pathogens. As observations of *mecB* in *S. aureus* are rare, the role of *mecB*-encoded PBP2a in conferring resistance to anti-MRSA cephalosporin antibiotics is underexplored. Here, we showed that acquiring the *mecB* gene leads to resistance to ceftobiprole and ceftaroline (Table S3), similar to introducing mutated *mecA* into the susceptible *S. aureus* background (51). However, the sequence coverage of the transferred plasmid p19Msa1099_1 was 24 times lower than the coverage of the entire *S. aureus* RN4220 chromosome. Also, the phenotypic test showed frequent plasmid losses at cell division. We hypothesize that the plasmid is not uniformly maintained in the bacterial cell population, and its segregation stability is low for unknown reasons (52). In addition to the *mecB* encoding plasmid, a 1.9 kb cryptic plasmid was transmitted to the analyzed transconjugants. Whether plasmid 19Msa1099_8 encodes for a function that is beneficial for the acquisition or maintenance of p19Msa1099_1 remains unknown.

Linear megaplasmids have been found in a limited number of bacterial species. These plasmids are extrachromosomal DNA elements that are divided into hairpin elements or elements with 5′-end attached proteins (53). Both types are characterized by an invertron-like structure with ITRs. The assembly of a complete plasmid p19Msa1099_9 was achieved by obtaining high-quality nanopore sequencing reads with lengths exceeding the ITRs. The ONT platform further enabled the sequencing of unwound plasmid strands that contained the DNA hairpin closure, allowing the identification of the junction site of both strands. As there were no obvious nicks in the sequence, a linear hairpin structure could be confirmed. Comparative analysis of the nucleotide sequences with other linear plasmids in the GenBank found no sequence similarity. However, at the protein level, a low similarity and similar gene structure with the linear plasmid of *Mammaliicoccus sciuri* strain B9-58B isolated from retail pork has been identified (54). The linear plasmid contains a large number of IS30-like elements (Fig. 8) that are widely distributed in bacteria and are involved in the transfer of genetic information and shuttling of adaptive traits and, thus, influences the genomic content (55). Predicted genes of possible replication relevance are scattered throughout the plasmid. We identified the two putative genes encoding the RepB family initiation protein (Fig. 8). RepB is a hexameric replication initiator typical for the rolling-circle replicons evolutionarily related to viral initiators, whereas other plasmid initiators are purified as monomers or dimers (56). The plasmid encodes the VirB4 ATPase, several putative hydrolases, and the protein containing the FtsK domain, which are part of the multiprotein secretion apparatus, the type IV secretion system (T4SS), that is necessary for the conjugative transfer of DNA to a recipient cell. VirB4 exhibits similarity to the TraE protein involved in the process of DNA translocation, FtsK-homologous protein transfers double-stranded DNA to the recipient cell, and hydrolases play a key role in the assembly of T4SS (34).

## MATERIALS AND METHODS

### Bacterial strains

Strains NRL/St 95/376^T^, NRL/St 13/116, and NRL/St 21/332 were isolated in various routine clinical laboratories in the Czech Republic and identified in the Reference Laboratory for Staphylococci (National Institute of Public Health, Prague, Czech Republic). Strains 19Msa1099, 19Msa1047, and 19Msa0499 were isolated previously (21). Representative strains have been deposited in the Czech Collection of Microorganisms (Masaryk University, Brno, Czech Republic, http://www.sci.muni.cz/ccm) and the Leibniz Institute DSMZ-German Collection of Microorganisms and Cell Cultures (Braunschweig, Germany, https://www.dsmz.de) (Table 1). Reference type strains of validly named *Macrococcus* spp. were obtained from the Czech Collection of Microorganisms.

### Phenotypic characterization

The morphological, biochemical, and physiological characterization of analyzed macrococci was performed as previously described (7, 57, 58). The growth of strains was tested in a basic set of staphylococcal media: Columbia sheep-blood Agar (Oxoid), Plate Count Agar (Oxoid), Tryptone Soya Agar (Oxoid), CM3 Nutrient Agar (Oxoid), Brain Heart Infusion Agar (Oxoid), Mannitol Salt Agar (HiMedia), and P agar at mesophile temperature conditions. The tested temperatures were 1°C, 3°C, 7°C, 10°C, 15°C, 25°C, 37°C, 42°C, 45°C, 48°C, and 50°C. The tested salinity was 0%, 2%, 7%, 8%, 9%, 10%, and 11% NaCl in Tryptone Soy Broth (TSB) (Oxoid). The tested pH levels (in TSB) were 5, 6, 7, 8, 9, 10, and 11. The commercial kits API ZYM, API 50 CH (bioMérieux), and Biolog GEN III MicroPlate (Biolog) were used as specified by the manufacturers. Antimicrobial susceptibility was tested by the disc diffusion method using Oxoid disks except for ceftobiprole and ceftaroline, which were from Bio-Rad, on Mueller-Hinton agar and interpreted according to the EUCAST guidelines for coagulase-negative staphylococci (59) as described previously (60).

### Transmission electron microscopy

A 200-mesh carbon/formvar-coated grid was placed on a drop of suspension of bacteria in water for 20 min. Bacterial cells on the grid were negatively stained with 2% ammonium molybdate and treated with UV light. A Morgagni 268D Philips (ThermoFisher Scientific, The Netherlands) transmission electron microscope was used to visualize bacterial cells.

### Matrix-assisted laser desorption/ionization-time-of-flight mass spectrometry analysis

The samples were prepared by the standard extraction protocol described by Freiwald and Sauer (61). In addition to the standard saturated solution of alpha-cyano-4-hydroxycinnamic acid (CHCA) in water:acetonitrile:TFA (47.5:50:2.5, vol/vol), an alternative matrix solution was utilized, composed of 12.5 mg mL^−1^ ferulic acid in water:acetonitrile:formic acid (50:33:17, vol/vol), as proposed by Madonna et al. (25). MALDI-TOF mass spectra were obtained using an Ultraflextreme instrument (Bruker Daltonics) operated in linear positive mode using the software FlexControl v3.4. Mass spectra were processed using FlexAnalysis v3.4 (Bruker Daltonics) and BioTyper software v3.1 (Bruker Daltonics). A MALDI-TOF mass spectra-based dendrogram was constructed using the Pearson’s product moment coefficient as a measure of similarity, and the unweighted pair group average linked method (UPGMA) as a grouping method.

### Genotypic analysis by fingerprinting techniques

Repetitive sequence-based PCR (rep-PCR) fingerprinting with the (GTG)_5_ primer was used to genotypically screen the investigated macrococci. DNA extraction from the bacterial cells, PCR conditions, and fingerprint analysis were performed as previously described (62). Further characterization included automated ribotyping with the restriction enzyme *Eco*RI, performed with the RiboPrinter Microbial Characterisation System (DuPont Qualicon) according to the manufacturer’s instructions. Numerical analysis of rep-PCR fingerprints and ribotype patterns was performed using BioNumerics v7.6 (Applied Maths, Belgium). The dendrograms were calculated with Pearson’s correlation coefficients with the UPGMA clustering method. The ribotype patterns were imported into the software BioNumerics using the load samples import script provided by the manufacturer.

### Chemotaxonomic analyses

Analyses were performed in the proposed type strain NRL/St 95/376^T^ at the Leibniz Institute DSMZ-German Collection of Microorganisms and Cell Cultures (https://www.dsmz.de). Fatty acid methyl ester (FAME) profiles were analyzed after saponification, methylation, and extraction following the protocol of Sasser (63). The FAME mixtures were separated by gas chromatography and detected by a flame ionization detector. In subsequent analysis, fatty acids were identified by gas chromatography-mass spectrometry (GC-MS) run on an Agilent GC-MS 7000D system (64). The position of single double bonds was confirmed by derivatization to the corresponding dimethyl disulfide adduct (65). Peaks were identified based on retention time and mass spectra. If the MIDI database TSBA6 resulted in a different identification, it is mentioned in the Protologue to allow comparison to the literature.

Isolation and structure analyses of the peptidoglycan were performed according to published protocols with some modifications. In brief, the amino acid composition of the total hydrolysate (4 N HCl, 100°C, 16 h) of the peptidoglycan was analyzed by GC-MS using Protocol 10 by Schumann (66). The partial hydrolysate (4 N HCl, 100°C, 45 min) of the peptidoglycan was analyzed by high-resolution liquid chromatography-mass spectrometry as described previously (66, 67). Enantiomeric analysis was performed by liquid chromatography as described previously (68). Respiratory quinones were extracted and analyzed as described previously (64). Identity was confirmed by mass spectrometry as described previously (67).

### Conjugative transfer of methicillin resistance

The protocol for conjugation between donor *M. psychrotolerans* sp. nov. strain 19Msa1099 and recipient *S. aureus* RN4220 (pT181) (69) was based on the original method by Forbes and Schaberg (70) with the following modifications: The cultures were grown overnight at 37°C in TSB, diluted 1:50 in TSB, and grown with aeration at 37°C to OD_600_ = 0.4 (approximately 10^8^ CFU mL^−1^). The cultures were mixed 1:1 and filtered through a sterile 0.45 µm cellulose acetate filter (Ahlstrom-Munksjö, Finland). The filter was placed on a TSA plate without antibiotics, with the side with the bacterial lawn touching the agar surface, and incubated overnight at 37°C. The cells were washed from the filter into 1 mL of TSB, plated at various dilutions on TSA plates with 2.5 µg mL^−1^ tetracycline (Sigma-Aldrich) and 1.25 µg mL^−1^ oxacillin sodium salt (Sigma-Aldrich), and incubated overnight at 37°C. Individual tiny colonies were passaged on TSA plates with 2.5 µg mL^−1^ tetracycline and 0.25 µg mL^−1^ oxacillin.

For simple calculation of plasmid stability during cell replication, the culture grown on TSA with oxacillin 0.25 µg mL^−1^ was resuspended in TSB and diluted and plated on TSA without oxacillin to obtain individual colonies. The colonies were replicated on plates with oxacillin 0.25 µg mL^−1^, and plasmid stability was calculated as the ratio of the number of oxacillin-resistant colonies over the number of input colonies.

### Genome sequencing and assembly

The sequencing of strains 19Msa1099, 19Msa1047, and 19Msa0499 was performed in a previous study as described by Keller et al. (21). For strains NRL 95/St 376^T^, NRL/St 13/116, and NRL/St 21/332, the genomic DNA was extracted from pure cultures cultivated in Meat Peptone Broth as described previously (71). The 500 bp sequencing library for the Illumina platform was prepared with a NEBNext Ultra II DNA library prep kit for Illumina (New England BioLabs). The samples were sequenced using a MID output cartridge in 150 bp paired-end mode in an Illumina NextSeq sequencing platform (Illumina, San Diego, CA, USA). The quality of reads was assessed with FastQC v0.11.9 (72).

For sequencing using the Oxford Nanopore technology (ONT), the library was prepared using the SQK-RAD004 Rapid sequencing kit (Oxford Nanopore Technologies, Oxford, UK) according to the manufacturer’s instructions, and sequenced with a FLO-FLG001 flow cell (R9.4.1) in a MinION device (Oxford Nanopore Technologies, Oxford, UK). The device was controlled with the software MinKNOW v22.12.7 (Oxford Nanopore Technologies, UK) also used for base calling and trimming. To verify the genetic background of the transconjugants and for the linear megaplasmid assembly, the SQK-LSK114 Ligation sequencing kit and SQK-RBK114-24 Rapid barcoding kit (Oxford Nanopore Technologies) and FLO-FLG114 flow cells (R10.4.1) were used. The software MinKNOW v24.02.16 (Oxford Nanopore Technologies) was used for base calling and trimming. In all cases, only reads with a *q*-score ≥10 were used for assembly. Long sequencing reads were mapped to the reference chromosomal and plasmid sequences using the built-in Geneious Mapper (Minimum mapping quality: 10) in Geneious Prime v2024.0.5 (GraphPad Software, New Zealand).

Complete bacterial genome sequences were assembled using Trycycler v0.5.0 (73). The resulting assemblies were first polished with Medaka using long reads (https://github.com/nanoporetech/medaka) and then Polypolish using short reads (74). The coverage of the final assembly was inspected by mapping sequencing reads using the plugin module Minimap2 (75) for ONT reads and Bowtie 2 (76) for Illumina reads in Geneious Prime.

### Phylogenic and pangenome analyses

An almost complete 16S rRNA gene was sequenced by Sanger sequencing (Eurofins Genomics, Ebersberg, Germany) as described previously (77). The whole 16S rRNA gene sequences for phylogenetic analyses were extracted from WGS data obtained in this study and the NCBI Genome database and compared by EzBioCloud (78). The phylogenetic analyses were performed with the software MEGA v11 (79). Genetic distances were corrected using the Tamura-Nei model, and the evolutionary history was inferred using the maximum-likelihood and neighbor-joining methods, using a bootstrap test based on 500 replications (80). The up-to-date bacterial core gene (UBCG) pipeline v3.0 with default settings was used for whole-genome phylogenetic analysis based on 92 core gene sequences (81).

Average nucleotide identity (ANI) and digital DNA-DNA hybridization (dDDH) values were determined to evaluate the genomic similarity between strains. The dDDH values were calculated according to the d4 formula using a web-based genome-to-genome distance calculator (GGDC) v3.0 (82). Cluster analysis based on ANI values and a heat map of ANI identity were obtained using fastANI v1.33 (83).

### Bioinformatics analyses

Complete genomes were annotated using the NCBI Prokaryotic Genome Annotation Pipeline (84) and further examined manually with the NCBI BLAST tool (https://blast.ncbi.nlm.nih.gov). MGEs were identified with PHASTER (85), PhiSpy v4.2.21 (86), IslandViewer 4 (87), VRprofile2 (88), and ISFinder (89). Virulence and resistance genes were predicted using ABRicate (https://github.com/tseemann/abricate) with the CARD (90), Resfinder v4.5.0 (91), and VFDB (92) databases. Predicted protein sequences were clustered using web-based OrthoVenn3 with a default cutoff *e*-value of 1*e*−5 and inflation value of 1.5 (93). The pairwise sequence alignments of multiple loci and whole genomes were visualized using EasyFig v2.2.5 (94).

## Data Availability

All sequence data associated with the analyzed strains have been deposited in GenBank under BioProject accession numbers PRJNA952914 and PRJNA744395. The GenBank accession numbers of complete genomes of strains NRL/St 95/376^T^, NRL/St 13/116, and NRL/St 21/332 are CP124591-CP124597, CP124585-CP124590, and CP124577-CP124584, respectively. Whole genomic sequences of strains 19Msa1099 (CP079955-CP079964), 19Msa0499 (CP079969-CP079980), and 19Msa1047 (CP080013-CP080023) have been published previously (21). The ONT sequencing data from an *S. aureus* transconjugant have been deposited under BioSample accession number SAMN41884817. The partial 16S rRNA gene sequence of type strain NRL/St 95/376^T^ obtained by Sanger sequencing was deposited under GenBank accession number PP341307.

## SUPPLEMENTAL MATERIAL

Supplemental material aem.01652-24-s0001.xlsx Tables S1 to S7.

ASM does not own the copyrights to Supplemental Material that may be linked to, or accessed through, an article. The authors have granted ASM a non-exclusive, world-wide license to publish the Supplemental Material files. Please contact the corresponding author directly for reuse.
